# Blood Test Results of Pregnant COVID-19 Patients: An Updated Case-Control Study

**DOI:** 10.3389/fcimb.2020.560899

**Published:** 2020-10-07

**Authors:** Guoqiang Sun, Yizhi Zhang, Qing Liao, Yao Cheng

**Affiliations:** ^1^Obstetric Department, Maternal and Child Health Hospital of Hubei Province, Wuhan, China; ^2^Longquanyi District of Chengdu Maternal and Child Health Care Hospital, Chengdu, China

**Keywords:** COVID-19, blood lab test, case-control, pregnancy with hypertension, pregnancy with diabetes

## Abstract

**Background:** Coronavirus disease (COVID-19) is a current global public health emergency. However, current research on the blood test results of pregnant women with COVID-19 is insufficient.

**Methods:** A case-control study was carried out based on clinical blood test results. Pregnant COVID-19 patients, pregnant COVID-19 patients with diabetes, and pregnant COVID-19 patients with hypertension, were assessed in this study. Also, 120 controls were matched by age, parity, fetus number, and presence of chronic disease. *T*-tests, Chi-square tests, Wilcoxon signed-rank tests, and Kruskal-Wallis tests were used to compare data from the blood tests and liver function indices among the selected groups.

**Results:** Between January 24 and March 14, 2020, 60 pregnant COVID-19 patients delivered at the Maternal and Child Health Hospital of Hubei Province. The average maternal age of pregnant COVID-19 patients was 30.97 years and the mean gestational period was 37.87 weeks. 71.67% (43/60) of pregnant COVID-19 patients gave birth by cesarean delivery. In total, 21.67% (13/60) were diagnosed with diabetes and 18.33% (11/60) were diagnosed with hypertension during pregnancy. Compared to controls, pregnant COVID-19 patients showed significantly lower numbers of blood lymphocytes and higher numbers of neutrophils, as well as higher levels of C-reactive protein and total bilirubin. Among the three groups, pregnant COVID-19 patients with diabetes had significantly higher levels of neutrophils and lower levels of total protein. Aspartate transaminase levels were higher in pregnant COVID-19 patients with hypertension than in pregnant COVID-19 patients with no comorbidities and controls with hypertension.

**Interpretations:** Blood and liver function indices indicate that chronic complications, including hypertension and diabetes, could increase the risk of inflammation and liver injury in pregnant COVID-19 patients.

## Introduction

SARS-CoV-2, which causes coronavirus disease (COVID-19), is a new coronavirus discovered in 2019. It is similar to previously studied zoonotic viruses, including the severe acute respiratory syndrome coronavirus (SARS-CoV-1) and the Middle East respiratory syndrome coronavirus (MERS-CoV) (Liu Y. et al., 2020). Since its first report in Wuhan, China in December 2019, over 3.8 million people have been infected with SARS-CoV-2, and it has caused over 260,000 deaths worldwide (Rothan and Byrareddy, 2020; World Health Organization, 2020). The World Health Organization (WHO) declared the SARS-CoV-2 outbreak to be a public health emergency of international concern on January 30, 2020, indicating a high global risk (Sohrabi et al., 2020).

To provide practical evidence for the prevention and control of COVID-19, many studies have been conducted on the general population. However, pregnant women are more vulnerable to COVID-19 because they are in an immunocompromised state (Liang and Acharya, 2020). Pneumonia arising from any infectious etiology is an important cause of morbidity and mortality among pregnant women (Schwartz and Graham, 2020). In addition, both hypertension and diabetes are common complications during pregnancy, which have long-term negative health impacts on both mothers and their offspring (Davenport et al., 2018). Thus, pregnant women, especially those with chronic complications, should be evaluated as a high-risk group during the current COVID-19 pandemic (Liu D. et al., 2020).

To date, most studies (Chen H. et al., 2020; Chen N. et al., 2020; Schwartz, 2020) on pregnant women with COVID-19 have described their clinical characteristics including symptoms, pregnancy outcomes, CT manifestation, and mother-child vertical transmission. However, the indication for the adoption of cesarean delivery was controversial at the beginning of COVID-19 epidemic because parturient with COVID-19 might be associated with increased risk of miscarriage, preterm birth, and neonatal death (Boelig et al., 2020; Chen Z. et al., 2020; Wu et al., 2020). And there are few studies assessing pregnant women with COVID-19 who have comorbidities including hypertension and diabetes. The purpose of this study was to compare blood test results and liver function indices among pregnant COVID-19 patients and control groups of ordinary pregnant women.

## Methods

### Participants

Patients included in the study delivered at the Maternity and Child Health Hospital in Wuhan, Hubei province. In 2018, 22.55% of newborns in Wuhan were delivered at this hospital, making it the largest maternal hospital in Hubei. From January 24 to March 14, 2020, 3,730 pregnant women delivered in this hospital, and 60 of them (1.61%) were confirmed to have COVID-19 by laboratory and clinical diagnosis (Juan et al., 2020). Moreover, we retrospectively selected 120 controls matched by age (within 3 years), parity, fetus number, and chronic disease (including diabetes, hypertension, hypothyroidism, and hepatic disease). Control patients were selected 1 year before the COVID-19 epidemic. Sociodemographic information and blood samples were collected upon admission and results were obtained from medical health records. All research subjects were recruited in the third trimester.

### Data Analysis

Maternal and neonatal characteristics of continuous variables are presented as mean ± SD. *T*-tests, Chi-square tests, Wilcoxon signed-rank tests, and Kruskal-Wallis tests were used to compare the characteristics between the pregnant COVID-19 patients and control groups. Data analyses were conducted using the Statistical Analysis System (SAS 9.4). Two-sided *p* < 0.05 were considered statistically significant.

## Results

Sociodemographic characteristics of pregnant COVID-19 patients and control groups are shown in Table 1. The average age of the pregnant COVID-19 patients was 30.97 years and the mean gestational period was 37.87 weeks. These patients presented with mild COVID-19 infection. A high percentage of diabetes and hypertension was detected among the pregnant COVID-19 patients (21.67 and 18.34%, respectively). The majority of pregnant patients gave birth by cesarean delivery (71.67%), significantly higher than that of control groups (42.50%). Most pregnant COVID-19 patients showed no fever or respiratory symptoms, and only 15% reported respiratory symptoms during hospitalization. There were 61 live-birth newborns without COVID-19 infection. Moreover, Table S1 presents the characteristics of maternal COVID-19 patients with chronic disease. The difference of clinical symptoms showed no statistical significance between pregnant COVID-19 patients with chronic disease and COVID-19 patients without chronic disease. Cesarean delivery in COVID-19 patients with chronic was significantly higher than in COVID-19 patients without chronic disease (91.67 vs. 58.33%, *p* = 0.0054).

**Table 1:** Sociodemographic characteristics of pregnant COVID-19 patients and control group.

	**COVID-19 patients**	**Control patients**	***P***
	***N* (%)/*N* ± SD**	***N* (%)/*N* ± SD**	
Age	30.97 ± 4.13	29.97 ± 3.43	0.0871
**Parity**			
1	40 (66.67)	80 (66.67)	–
2	19 (31.67)	38 (31.67)	
3	1 (1.67)	2 (1.67)	
**Fetus number**			
1	59 (98.33)	118 (98.33)	–
2	1 (1.67)	2 (1.67)	
**Chronic disease**			
None	36 (60.00)	72 (60.00)	–
Diabetes	10 (16.67)	20 (16.67)	
Hypertension	8 (13.33)	16 (13.34)	
Diabetes and hypertension	3 (5.00)	6 (5.00)	
Hypothyroidism	2 (3.33)	4 (3.33)	
Hepatic disease	1 (1.67)	2 (1.67)	
Gestational week of delivery	37.87 ± 2.36	38.81 ± 1.81	0.0078
**Gestational week of delivery**			
<37	10 (16.67)	9 (7.50)	0.0599
≥37	50 (83.33)	111 (92.50)	
**Delivery**			
Vaginal delivery	17 (28.33)	69 (57.50)	0.0002
Cesarean delivery	43 (71.67)	51 (42.50)	
**First symptoms**			
None	26 (28.33)	0	–
Fever before or during delivery	11 (18.33)	0	
Fever after delivery	23 (38.33)	0	
Respiratory symptoms	9 (15.00)	0	
Neonatal birthweight (g)	3220 ± 622	3251 ± 520	0.7247
**1-min Apgar scores**			
10	38 (63.33)	14 (58.33)	0.5152
<10	22 (36.67)	10 (41.67)	
**5-min Apgar scores**			
10	57 (95.00)	23 (95.83)	0.8105
<10	3 (5.00)	1 (4.17)	

Blood test results of the study groups are presented in Table 2. There was a statistically significant difference in the number of blood lymphocytes, neutrophils, and C-reactive protein (CRP) between pregnant COVID-19 patients and control women. Moreover, blood total bilirubin (TBil) levels in pregnant COVID-19 patients were significantly higher than those in controls (8.25 vs. 6.57, *p* < 0.05). The results of Chi-square analysis further confirmed that the proportion of lymphocytopenia among COVID-19 patients was significantly higher than control groups (43.33 vs. 15.83%, *p* = 0.0003) (Table S2). The proportion of COVID-19 patients with elevated CRP was higher than the control groups (60.00 vs. 38.33%, *p* = 0.0061). The blood test results of liver function showed that the proportion of COVID-19 patients with elevated aspartate aminotransferase (AST) was higher than the ordinary patients (8.33 vs. 0.83%, *p* = 0.0084). Moreover, the proportion of COVID-19 patients with decreased blood TBil level was lower than the control groups (11.67 vs. 29.17%, *p* = 0.0091).

**Table 2:** Blood test results of pregnant COVID-19 patients and control group.

	**COVID-19 patients**		**Control patients**		***P***
	**Mean**	**SD**	**Mean**	**SD**	
WBC, 10^9^/L	10.68	3.87	9.60	3.00	0.0771
Lymphocytes, 10^9^/L	**1.25**	**0.53**	**1.66**	**1.18**	**0.0001**
Neutrophils, 10^9^/L	**80.83**	**8.88**	**77.17**	**6.10**	**0.0051**
CRP, mg/L	**20.16**	**41.96**	**8.87**	**20.41**	**0.0015**
ALT, U/L	15.07	25.21	10.03	4.81	0.7200
AST, U/L	22.33	26.81	16.59	5.00	0.1796
TBil, umol/L	**8.25**	**3.24**	**6.57**	**2.81**	**0.0010**
TP, g/L	66.79	4.70	66.91	4.78	0.8843
ALB, g/L	35.86	3.03	35.78	3.40	0.9853
GLB, g/L	30.93	3.63	31.43	4.15	0.5720

*WBC, white blood cell; CRP, C-reactive protein; ALT, alanine aminotransferase; AST, aspartate aminotransferase; TBil, total bilirubin; TP, total protein; ALB, albumin; GLB, globulin. Bold represented statistical significance*.

Blood test results of pregnant COVID-19 patients and controls with diabetes are presented in Table 3. The white blood cell (WBC) count of pregnant COVID-19 patients without diabetes was significantly higher than that in the pregnant controls with diabetes (10.20 vs. 8.49, *p* < 0.05). The number of neutrophils in pregnant COVID-19 patients with diabetes was higher than that in both pregnant COVID-19 patients without chronic disease and controls with diabetes (80.98 vs. 78.67 and 75.61, respectively, *p* < 0.05). The TBil level in COVID-19 patients without diabetes was higher than that in pregnant controls with diabetes (7.23 vs. 5.93, *p* < 0.05). Finally, total protein (TP) levels in the pregnant COVID-19 patients with diabetes were significantly lower than that in the controls with diabetes (64.64 vs. 68.52, *p* < 0.05). Table S3 shows that the proportion of participants with decreased albumin (ALB) was higher than the ordinary patients with diabetes (46.15 vs. 19.23%, *p* = 0.042).

**Table 3:** Blood test results of pregnant COVID-19 patients and control pregnant patients with diabetes.

	**COVID-19 patients**		**COVID-19 patients**		**Control patients**	
	**with diabetes**		**without diabetes**		**with diabetes**	
WBC, 10^9^/L	9.95	3.08	**10.20**	**3.38**	**8.49**	**2.16**
Lymphocytes, 10^9^/L	1.22	0.43	1.57	1.16	1.49	0.36
Neutrophils, 10^9^/L	**80.98**	**7.73**	**78.67**	**7.56**	**75.61**	**4.75**
CRP, mg/L	10.61	22.96	13.65	28.64	3.60	1.77
ALT, U/L	14.45	10.08	11.69	17.16	9.87	4.42
AST, U/L	19.79	10.77	18.67	18.20	16.16	3.41
TBil, umol/L	7.76	3.74	**7.23**	**3.11**	**5.93**	**1.83**
TP, g/L	**64.64**	**3.74**	66.69	4.80	**68.52**	**4.51**
ALB, g/L	35.48	2.76	35.61	3.40	36.92	2.83
GLB, g/L	29.17	3.28	31.35	4.18	31.60	3.38

*WBC, white blood cell; CRP, C-reactive protein; ALT, alanine aminotransferase; AST, aspartate aminotransferase; TBil, total bilirubin; TP, total protein; ALB, albumin; GLB, globulin. Bold represented statistical significance*.

Blood test results of pregnant COVID-19 patients and controls with hypertension are presented in Table 4. WBC, lymphocytes, neutrophils, and CRP were unchanged across the different groups. In contrast, AST levels in pregnant COVID-19 patients with hypertension were significantly higher than that in pregnant COVID-19 patients without chronic disease and controls with hypertension (38.97 vs. 16.61 and 19.09, respectively, *p* < 0.05). Additionally, ALB levels in pregnant COVID-19 patients with hypertension were significantly lower than that in pregnant COVID-19 patients without hypertension (33.67 vs. 36.08, *p* < 0.05). Table S4 shows that the proportion of lymphocytopenia of COVID-19 patients with hypertension was significantly higher than that of control patients with hypertension (18.18% vs. 9.09, *p* = 0.049).

**Table 4:** Blood test results of pregnant COVID-19 patients and control pregnant patients with hypertension.

	**COVID-19 patients**		**COVID-19 patients**		**Control patients**	
	**with hypertension**		**without hypertension**		**with hypertension**	
WBC, 10^9^/L	8.95	2.39	9.87	3.11	10.72	4.29
Lymphocytes, 10^9^/L	1.39	0.36	1.54	1.14	1.55	0.46
Neutrophils, 10^9^/L	77.10	8.91	78.59	7.17	77.68	7.57
CRP, mg/L	26.01	59.39	10.16	20.77	15.66	29.94
ALT, U/L	28.56	51.17	10.62	8.70	9.39	3.87
AST, U/L	**38.97**	**56.56**	**16.61**	**7.30**	**19.09**	**5.28**
TBil, umol/L	7.25	2.18	7.19	3.18	6.22	2.29
TP, g/L	65.10	4.68	67.03	4.47	66.35	6.28
ALB, g/L	**33.67**	**2.89**	**36.08**	**2.94**	35.09	4.92
GLB, g/L	31.44	2.68	30.95	3.23	32.85	7.53

*WBC, white blood cell; CRP, C-reactive protein; ALT, alanine aminotransferase; AST, aspartate aminotransferase; TBil, total bilirubin; TP, total protein; ALB, albumin; GLB, globulin. Bold represented statistical significance*.

## Discussion

This is an updated case-control study comparing the blood test results of pregnant women with COVID-19. Among the pregnant COVID-19 patients, there were 43 of 60 cases (71.67%) underwent cesarean section. This result is in line with previous studies which reported a range of 56–93% of cesarean section (Chen L. et al., 2020; Huntley et al., 2020; Walker et al., 2020; Yang et al., 2020). The high adoption of cesarean delivery might be partly explained by the COVID-19 infection combined with the presents of chronic disease and the concern about the effects of COVID-19 on the pregnancy. We found that the most prevalent symptom of pregnant COVID-19 patients was fever, and a majority had no respiratory symptoms. These findings are consistent with other studies (Chen L. et al., 2020; Guan et al., 2020). Zaigham and Andersson (2020) also reported that most COVID-19 patients did not have any symptoms upon admission. Therefore, it is necessary to separate COVID-19 infected patients from ordinary patients by laboratory indicators upon hospital admission. Moreover, maternal symptoms of COVID-19 during the first or second trimester of pregnancy require further research.

This study showed a high percentage of diabetes and hypertension among pregnant COVID-19 patients. A previous study based on 30 pregnant COVID-19 patients confirmed that the most common comorbidities were hypertension (16.7%) and diabetes (6.6%) (Wang et al., 2020). The associations between COVID-19 infection and the development of diabetes and hypertension among pregnant women were still unclear. The potential mechanism might be explained by the expression of Angiotensin-converting-enzyme 2 (ACE2), the receptor for the COVID-19 spike protein, on pancreatic β cells can have a direct effect on β cell function, which might result in the development of diabetes among pregnant COVID-19 patients (Bornstein et al., 2020). Moreover, the hyper-inflammatory state in COVID-19 may be associated with a higher risk of developing pre-eclampsia (Abbas et al., 2020).

As far as we know, this is the first study to explore the blood test results of pregnant women with chronic disease who are diagnosed with COVID-19. Compared to the pregnant women without COVID-19, the blood indices of pregnant COVID-19 patients showed a significantly lower lymphocyte count than the controls. Other indices including neutrophil count and CRP levels of the pregnant COVID-19 patients were significantly higher than those of the control pregnant women. These differences in inflammatory indices are in accordance with typical clinical characteristics of pneumonia. Chen L. et al. (2020) reported that lymphopenia was present in 51 of 116 pregnant women diagnosed with COVID-19, while. Liu D. et al. (2020) found that among 15 pregnant COVID-19 patients, 12 had a decreased lymphocyte count and 10 had increased CRP values. It is recommended that the blood indices of pregnant COVID-19 patients should be carefully monitored and the changes in these inflammatory indices were correlated with the prognosis of patients (Chen L. et al., 2020). The long-term effects of the COVID-19 infection and treatment on blood indices of pregnant COVID-19 patients need further study.

Additionally, pregnant women with COVID-19 showed significantly higher TBil values. Elevated TBil levels are considered biomarkers for liver disease (Weaver et al., 2018). Thus, COVID-19 could affect the liver function of pregnant women. However, there was no difference in ALT and AST levels in pregnant women with COVID-19. A cohort study (Guan et al., 2020) containing 1,099 COVID-19 cases showed that 10.5% of patients presented with abnormal bilirubin, 21.3% with ALT elevation, and 22.2% with AST elevation. It has been reported that 2–11% of patients with COVID-19 have liver comorbidities and 14–53% have abnormal levels of AST and ALT during disease progression (Zhang et al., 2020). The limited number of pregnant COVID-19 patients in this study may limit the power to detect changes in ALT and AST. Moreover, the majority of the COVID-19 patients in this study had a mild infection and liver injury was not obvious. Therefore, liver damage caused by COVID-19 during pregnancy is still unclear and needs further research.

In our study, pregnant COVID-19 patients with chronic diseases, including diabetes or hypertension, showed a stronger inflammation response and markers of liver injury compared to pregnant COVID-19 patients without chronic diseases and control pregnant women with chronic diseases. Compared to the other two groups, pregnant COVID-19 patients with diabetes had significantly higher neutrophil counts and lower TP values. The main feature of diabetes is hyperglycemia, which can increase the oxidative stress response and enhance the inflammatory response. Diabetes could also compromise the ability of the liver to regenerate (Mendes-Braz and Martins, 2018). Insulin resistance in diabetes influences liver cell apoptosis and causes liver dysfunction (Schattenberg and Schuchmann, 2009). Hajam and Rai (2019) found that diabetic rats had a significant increase in lipid peroxidation (LPO) in the liver, while ALT, AST, and ALP levels were significantly decreased in the antioxidative enzymatic system. Thus, under the combined effects of COVID-19 and diabetes, suboptimal inflammatory processes and liver function biomarkers might indicate a worse prognosis in pregnant women.

Moreover, pregnant COVID-19 patients with hypertension showed a significantly higher level of blood AST, which is an important indicator of liver function (Sookoian and Pirola, 2015). Arima et al. (2014) reported that hypertension exacerbated liver injury and hepatic fibrosis in spontaneously hypertensive rats. Han et al. (2015) revealed that trophoblastic mitochondrial damage may result in liver injury in preeclampsia-like mouse models. Therefore, it was hypothesized that pregnant COVID-19 patients with hypertension are more likely to suffer from liver injury than the other groups. However, few studies have focused on liver injury caused by hypertension and this hypothesis requires further verification.

Our study has several limitations. First, pregnant women with COVID-19 were only enrolled during their third trimester. The effects of COVID-19 on pregnant women in their first or second trimester are not clear. Second, this study only assessed pregnant COVID-19 patients during hospitalization, but long-term effects on the liver and other organs as well as newborns require further research. Third, we only compared the blood indices of pregnant COVID-19 patients with hypertension and diabetes. The mechanisms underlying the effects of COVID-19 on these parameters also need further exploration.

## Conclusion

The results of this study indicate that chronic diseases may strengthen the inflammatory response to COVID-19 and mediate liver damage in pregnant women. Therefore, during the treatment of pregnant women with COVID-19, focusing on inflammation and liver injury is necessary, especially for those with chronic diseases.

## Data Availability Statement

The datasets generated for this article are not readily available because The application of the raw data should be approved by the Ethics Committee of Maternal and Child Health Hospital of Hubei Province. Requests to access the datasets should be directed to Yao Cheng, chengyao2014@sina.com.

## Ethics Statement

The studies involving human participants were reviewed and approved by Medical Ethics Committee of Maternal and Child Health Care Hospital of Hubei Province. Written informed consent for participation was not required for this study in accordance with the national legislation and the institutional requirements.

## Author Contributions

GS, YC, and QL: wrote the manuscript. GS, YC, and YZ: conceived and designed the experiments. YC: analyzed the data and performed the experiments. All of the authors: gave final approval of the version to be submitted and agree to be accountable for all aspects of the work.

## Conflict of Interest

The authors declare that the research was conducted in the absence of any commercial or financial relationships that could be construed as a potential conflict of interest.

## Abbreviations

CRP: C-reactive protein
TBil: total bilirubin
TP: total protein
ALT: alanine aminotransferase
AST: aspartate aminotransferase
WBC: white blood cell
ALB: albumin
GLB: globulin.
