# Supplementary material for: Blood Test Results of Pregnant COVID-19 Patients: An Updated Case-Control Study
Source: Front Cell Infect Microbiol. 2020 Oct 7;10:560899. doi: 10.3389/fcimb.2020.560899 (PMC7575733; doi:10.3389/fcimb.2020.560899)
Supplement: Supplementary file 1 [file Data_Sheet_1.pdf]

Table S1. Sociodemographic characteristics of pregnant COVID-19 patients with chronic disease

	COVID-19 patients with chronic disease	COVID-19 patients without chronic disease	*P*
	N (%)/ N ±SD	N (%)/ N ±SD	
Age	31.00 ±3.83	30.92 ±4.61	0.9397
Parity			
1	19 (79.17)	21 (58.33)	-
2	5 (20.83)	14 (38.89)	
3	0	1 (2.78)	
fetus number			
1	23 (95.83)	36 (100.00)	-
2	1 (4.17)	0	
Gestational week of delivery	37.67 ±2.12	38.00 ±2.53	0.5964
Gestational week of delivery			
<37	5 (20.83)	5 (13.89)	0.4832
≥37	19 (79.17)	31 (86.11)	
Delivery			
Vaginal delivery	2 (8.33)	15 (41.67)	0.0054
Cesarean delivery	22 (91.67)	21 (58.33)	
First symptoms			
None	12 (50.00)	14 (38.89)	0.3988
Fever before or during delivery	4 (16.67)	7 (19.44)	0.7871
Fever after delivery	8 (33.33)	15 (41.67)	0.5189
Respiratory symptoms	4 (16.67)	5 (13.89)	0.7697
Neonatal birthweight (g)	3204 ±609	3230 ±639	0.8760
1-minute Apgar scores			
10	14 (58.33)	24 (66.67)	0.5152
<10	10 (41.67)	12 (33.33)	
5-minute Apgar scores			
10	23 (95.83)	34 (94.44)	0.8105
<10	1 (4.17)	2 (5.56)	

Table S2. Blood test results of pregnant COVID-19 patients and control group

	COVID-19 patients		Control patients		***P***
	**N**	**%**	**N**	**%**	
WBC, 10^9/L					
3.5-9.5	30	50.00	66	55.00	0.5273
>9.5	30	50.00	54	45.00	
Lymphocytes, 10^9/L					
<1.1	26	43.33	19	15.83	0.0003
1.1-3.2	33	55.00	97	80.83	
>3.2	1	1.67	4	3.33	
Neutrophils, 10^9/L					
40-75	13	21.67	40	33.33	0.1064
>75	47	78.33	80	66.67	
CRP, mg/L					
<4	24	40.00	74	61.67	0.0061
≥4	36	60.00	46	38.33	
ALT, U/L					
0-35	58	96.67	120	100.00	-
>35	2	3.33	0	0	
AST, U/L					
0-35	55	91.67	119	99.17	0.0084
>35	5	8.33	1	0.83	
TBil, umol/L					
<5	7	11.67	35	29.17	0.0091
5-21	53	88.33	85	70.83	
TP, g/L					
<66	25	41.67	50	41.67	-
66-83	35	58.33	70	58.33	
ALB, g/L					
<35	24	40.00	47	39.17	0.9144
35-52	36	60.00	73	60.83	
GLB, g/L					
<20	1	1.67	0	0	-
20-40	59	98.33	119	99.17	
>40	0	0	1	0.83	

Abbreviations: WBC: white blood cell; CRP: C-reactive protein; ALT: alanine aminotransferase; AST: aspartate aminotransferase; TBil: total bilirubin; TP: total protein; ALB: albumin; GLB: globulin.

Table S3. Blood test results of pregnant COVID-19 patients and control pregnant patients with diabetes

	COVID-19 patients with diabetes		COVID-19 patients without diabetes		Control patients with diabetes		*P*
	N	%	N	%	N	%	
WBC, 10^9/L							
3.5-9.5	8	61.54	72	51.06	16	61.54	0.2703
>9.5	5	38.46	69	48.94	10	38.46	
Lymphocytes, 10^9/L							
<1.1	6	46.15	36	25.53	3	11.54	0.3115
⩾1.1	7	53.85	105	74.47	23	88.46	
Neutrophils, 10^9/L							
40-75	3	23.08	39	27.66	11	42.31	0.1849
>75	10	76.92	102	72.34	15	57.69	
CRP, mg/L							
<4	6	46.15	76	53.90	16	61.54	0.5888
≥4	7	53.85	65	46.10	10	38.46	
ALT, U/L							
0-35	13	100.00	139	98.58	26	100.00	-
>35	0	0	2	1.42	0	0	
AST, U/L							
0-35	11	84.62	137	97.16	26	100.00	-
>35	2	15.38	4	2.84	0	0	
TBil, umol/L							
<5	2	15.38	32	22.70	8	30.77	0.4897
5-21	11	84.62	109	77.30	18	69.23	
TP, g/L							
<66	7	53.85	61	43.26	7	26.92	0.2032
66-83	6	46.15	80	56.74	19	73.08	
ALB, g/L							
<35	6	46.15	60	42.55	5	19.23	0.0420
35-52	7	53.85	81	57.45	21	80.77	
GLB, g/L							
<20	0	0	1	0.71	0	0	-
20-40	13	100.00	139	98.58	26	100.00	
>40	0	0	1	0.71	0	0	

Abbreviations: WBC: white blood cell; CRP: C-reactive protein; ALT: alanine aminotransferase; AST: aspartate aminotransferase; TBil: total bilirubin; TP: total protein; ALB: albumin; GLB: globulin.

Table S4. Blood test results of pregnant COVID-19 patients and control pregnant patients with hypertension

	COVID-19 patients with hypertension		COVID-19 patients without hypertension		Control patients with hypertension		***P***
	**N**	**%**	**N**	**%**	**N**	**%**	
WBC, 10^9/L							
3.5-9.5	7	63.64	82	55.78	7	31.82	0.0662
>9.5	4	36.36	65	44.22	15	68.18	
Lymphocytes, 10^9/L							
<1.1	2	18.18	41	27.89	2	9.09	0.0490
≥1.1	9	81.82	106	72.11	20	90.91	
Neutrophils, 10^9/L							
40-75	4	36.36	42	28.57	7	31.82	0.6630
>75	7	63.64	105	71.43	15	68.18	
CRP, mg/L							
<4	4	36.36	84	57.14	10	45.45	0.1892
≥4	7	63.64	63	42.86	12	54.55	
ALT, U/L							
0-35	10	90.91	146	99.32	22	100.00	-
>35	1	9.09	1	0.68	0	0	
AST, U/L							
0-35	9	81.82	143	97.28	22	100.00	-
>35	2	18.18	4	2.72	0	0	
TBil, umol/L							
<5	1	9.09	36	24.49	5	22.73	0.6335
5-21	10	90.91	111	75.51	17	77.27	
TP, g/L							
<66	6	54.55	58	39.46	11	50.00	0.2567
66-83	5	45.45	89	60.54	11	50.00	
ALB, g/L							
<35	8	72.73	53	36.05	10	45.45	0.1559
35-52	3	27.27	94	63.95	12	54.55	
GLB, g/L							
<20	0	0	1	0.68	0	0	-
20-40	11	100.00	146	99.32	21	95.45	
>40	0	0	0	0	1	4.55	

Abbreviations: WBC: white blood cell; CRP: C-reactive protein; ALT: alanine aminotransferase; AST: aspartate aminotransferase; TBil: total bilirubin; TP: total protein; ALB: albumin; GLB: globulin.
